# Supplementary material for: From Recharge, to Groundwater, to Discharge Areas in Aquifer Systems in Quebec (Canada): Shaping of Microbial Diversity and Community Structure by Environmental Factors
Source: Genes (Basel). 2022 Dec 20;14(1):1. doi: 10.3390/genes14010001 (PMC9858702; doi:10.3390/genes14010001)
Supplement: Supplementary file 1 [file genes-14-00001-s001.zip › Supplementary Material text.pdf]

**Median sequencing depth**

```
# use ASV table called arc
> library(phyloseq)
> library(microbiome)
> rowSums(arc)
> ps = phyloseq(otu_table(t(arc), taxa_are_rows=TRUE))
> total=median(sample_sums(ps))
> standf = function(x, t=total) round(t * (x / sum(x)))
> phyloseq_object_rar = transform_sample_counts(ps, standf)
> otu_tab_median <- t(abundances(phyloseq_object_rar))
> rowSums(otu_tab_median)
```

**Test for differences in the diversity indices**

```
# use a table with the Shannon diversity indices and the environmental variables called arcdiv
> library(FSA)
> dunnTest(Shannon~Habitat, data=arcdiv)
```

**Correlation between beta diversity matrix and environmental variables (PERMANOVA)**

```
# use the ASV table obtained after application of median sequencing depth called arcr and the environmental variable table called env
> library(vegan)
> adonis2(arcr~Habitat+Site, data=env, permuations=999, method="bray")
```

**Correlation between beta diversity matrix and environmental variables (db-RDA and variance partitioning)**

```
# Hellinger transform the ASV table called arcr
> library(vegan)
> arcrh=decostand(arcr, method="hell")

# use the Hellinger transformed ASV table called arcrh and the environmental variable table called env to test for correlation with a db-RDA
> darc=capscale(arcrh~ DOC + NH4 + pH + temp + DO + Bac1 + Bac2, env, dist="bray")
> anova(dnbac, by="terms", permu=200)

# estimate the implication of the significant variables
> varpart(Y=arcrh, X=~ NH4, ~temp, data=env)
```
