# Supplementary material for: From Recharge, to Groundwater, to Discharge Areas in Aquifer Systems in Quebec (Canada): Shaping of Microbial Diversity and Community Structure by Environmental Factors
Source: Genes (Basel). 2022 Dec 20;14(1):1. doi: 10.3390/genes14010001 (PMC9858702; doi:10.3390/genes14010001)
Supplement: Supplementary file 1 [file genes-14-00001-s001.zip › Table S1.pdf]

Table S1. Sequence statistics for the archaeal and bacterial 16S rRNA reads.

Archaea

Sample	Number of sequences after cleaning	Number of sequences after median sequencing depth
V_soil_Jul18	193	na
V_soil_May19	10,337	28,433
SL_soil_Jul18	100	na
SL_soil_May19	8,778	27,096
CH_peat_Aug18	107,017	23,780
K1_soil_Aug18	24,434	26,987
K2_soil_Aug18	20	na
RR_soil_Jul19	12	na
ND_soil_Jul19	5,047	26,591
V_snow_Feb19	13,847	27,447
SL_snow_Feb19	2,746	27,400
V_gw_Jul18	58,223	22,514
V_gw_Feb19	17,898	28,625
V_gw_Apr19	70,179	21,804
V_gw_May19	111,178	22,560
SL_gw_Jul18	2,739	27,387
SL_gw_May19	74,466	24,736
CH_gw_Aug18	62,342	21,901
K1_gw_Aug18	1,411	27,287
K2_gw_Aug18	74,020	24,152
RR_gw_Jul19	63,072	22,509
ND_gw_Jul19	12,829	26,514
SL_dis1_Jul18	27,385	27,385
SL_dis2a_Jul18	19,440	25,377
SL_dis2b_Jul18	21,907	26,006
V_riv_Jul18	43,216	29,229
V_riv_Apr19	40,386	29,068
V_riv_May19	97,678	23,624
SL_riv_Jul18	51,479	31,013
SL_riv_May19	186,689	21,909
CH_stream_Aug18	15,566	27,908
K1_riv_Aug18	26,662	26,953
K2_riv_Aug18	29,643	27,900
RR_riv_Jul19	44,468	32,118
ND_lake_Jul19	11,038	26,088

Bacteria

Sample	Number of sequences after cleaning	Number of sequences after median sequencing depth
V_soil_Jul18	111,143	33,357
V_soil_May19	7,182	34,467
SL_soil_Jul18	3,740	33,005
SL_soil_May19	6,572	32,867
CH_peat_Aug18	72,166	30,686
K1_soil_Aug18	16,432	32,870
K2_soil_Aug18	88	na
RR_soil_Jul19	5,040	33,602
ND_soil_Jul19	7,109	33,498

V_snow_Feb19	60,818	35,736
SL_snow_Feb19	133,828	30,680
V_gw_Jul18	55,018	33,935
V_gw_Feb19	63,480	34,958
V_gw_Apr19	67,415	29,378
V_gw_May19	89,547	29,219
SL_gw_Jul18	42,562	33,184
SL_gw_May19	59,627	35,622
CH_gw_Aug18	40,296	33,964
K1_gw_Aug18	129,302	32,401
K2_gw_Aug18	47,703	34,156
RR_gw_Jul19	12,703	33,507
ND_gw_Jul19	11,240	33,375
SL_dis1_Jul18	16,912	33,419
SL_dis2a_Jul18	20,217	34,575
SL_dis2b_Jul18	15,981	32,429
V_riv_Jul18	41,667	34,423
V_riv_Apr19	76,954	27,895
V_riv_May19	81,915	27,563
SL_riv_Jul18	47,151	34,362
SL_riv_May19	87,303	26,908
CH_stream_Aug18	18,180	33,567
K1_riv_Aug18	25,637	31,215
K2_riv_Aug18	18,347	34,567
RR_riv_Jul19	15,895	32,599
ND_lake_Jul19	10,448	32,426
