# Supplementary material for: From Recharge, to Groundwater, to Discharge Areas in Aquifer Systems in Quebec (Canada): Shaping of Microbial Diversity and Community Structure by Environmental Factors
Source: Genes (Basel). 2022 Dec 20;14(1):1. doi: 10.3390/genes14010001 (PMC9858702; doi:10.3390/genes14010001)
Supplement: Supplementary file 1 [file genes-14-00001-s001.zip › Table S2.pdf]

Table S2. Environmental variables used to run a correlation analysis between the archaeal and bacterial community composition and explanatory factors using db-RDA. DOC, dissolved organic carbon; DO, dissolved oxygen; Arc1, archaeal community composition represented by the first axis of a PCoA; Arc2, archaeal community composition represented by the second axis of a PCoA; Bac1, bacterial community composition represented by the first axis of a PCoA; Bac2, bacterial community composition represented by the second axis of a PCoA; V, Vaudreuil; SL, Saint-Lazare; CH; Covey Hill; K; Kenauk; RR, Rivière Rouge; ND, Notre-Dame-des-Laus; gw, groundwater.

**Sample**	**DOC**	**NH4**	**pH**	**Temp**	**DO**	**Bac1**	**Bac2**	**Arc1**	**Arc2**
	mg/L	mg/L		°C	%				
V_gw_Jul18	0.99	0,000	6.1	7.56	6.3	0.130949	-0.238791	-0.512189	0.066855
V_gw_Feb19	1.137	0,000	5.15	5.26	36.3	0.080967	-0.174359	-0.523668	0.070975
V_gw_Apr19	2.825	0,028	7.2	3.8	103.3	0.128533	-0.240541	-0.508457	0.058852
V_gw_May19	2.75	0,015	7.47	3.65	43.3	0.095332	-0.209736	-0.375605	0.021225
SL_gw_Jul18	0.43	0,000	7.5	6.43	100	0.102874	0.054045	0.147483	0.447770
SL_gw_May19	1.6	0,011	8.53	6.37	107	0.018147	-0.027610	0.105474	0.382553
CH_gw_Aug18	30.53	0,058	5.89	13.88	82.3	0.018115	-0.008918	0.058259	-0.002173
K1_gw_Aug18	5.17	0,075	7.46	10.49	14.5	-0.012429	-0.066595	0.016933	0.021696
K2_gw_Aug18	0.82	0,092	6.48	10.97	21.2	-0.004814	-0.027961	0.055366	-0.044974
RR_gw_Jul19	0.01	0,012416	6.23	10.1	0.75	0.003508	-0.077124	0.025611	0.019976
ND_gw_Jul19	9.9	0,310635	7.02	8.8	13.5	0.003087	-0.067104	0.025503	0.019854
