# Supplementary material for: From Recharge, to Groundwater, to Discharge Areas in Aquifer Systems in Quebec (Canada): Shaping of Microbial Diversity and Community Structure by Environmental Factors
Source: Genes (Basel). 2022 Dec 20;14(1):1. doi: 10.3390/genes14010001 (PMC9858702; doi:10.3390/genes14010001)
Supplement: Supplementary file 1 [file genes-14-00001-s001.zip › Table S3.pdf]

Table S3. Amova analysis for the 3 groups (CA1, CA2, and CA3 for the Archaea, and CB1, CB2, and CB3 for the Bacteria) identified in the PCoA plot.

Archaea

CA1-CA2	Among	Within	Total
SS	1.09575	1.79218	2.88793
df	1	6	7
MS	1.09575	0.298696	

Fs: 3.66844
p-value: 0.038

CA1-CA3	Among	Within	Total
SS	0.939161	3.99863	4.93779
df	1	10	11
MS	0.939161	0.399863	

Fs: 2.34871
p-value: 0.004

CA2-CA3	Among	Within	Total
SS	1.08658	3.77933	4.86591
df	1	10	11
MS	1.08658	0.377933	

Fs: 2.87506
p-value: 0.003

Bacteria

CB1-CB2	Among	Within	Total
SS	0.737508	2.41385	3.15136
df	1	6	7
MS	0.737508	0.402309	

Fs: 1.83319
p-value: 0.031

CA2-CA3	Among	Within	Total
SS	0.760516	4.3731	5.13361
df	1	10	11
MS	0.760516	0.43731	

Fs: 1.73908
p-value: 0.002

CA1-CA3	Among	Within	Total
SS	0.754024	4.36098	5.11501
df	1	10	11
MS	0.754024	0.436098	

Fs: 1.72902
p-value: 0.001
