# Supplementary material for: From Recharge, to Groundwater, to Discharge Areas in Aquifer Systems in Quebec (Canada): Shaping of Microbial Diversity and Community Structure by Environmental Factors
Source: Genes (Basel). 2022 Dec 20;14(1):1. doi: 10.3390/genes14010001 (PMC9858702; doi:10.3390/genes14010001)
Supplement: Supplementary file 1 [file genes-14-00001-s001.zip › Table S4.pdf]

Table S4. Correlation between the archaeal and bacterial community composition and explanatory factors using db-RDA. DOC, dissolved organic carbon; DO, dissolved oxygen; Arc1, archaeal community composition represented by the first axis of a PCoA; Arc2, archaeal community composition represented by the second axis of a PCoA; Bac1, bacterial community composition represented by the first axis of a PCoA; Bac2, bacterial community composition represented by the second axis of a PCoA.

	**Archaea**				**Bacteria**			
	**Df**	**SumOfSqs**	**F**	**Pr(>F)**	**Df**	**SumOfSqs**	**F**	**Pr(>F)**
**DOC**	1	0.49338	1.2732	0.088	1	0.49075	1.1705	0.041
**NH4**	1	0.55260	1.4260	0.010	1	0.53506	1.2761	0.003
**pH**	1	0.52177	1.3465	0.058	1	0.50370	1.2013	0.029
**Temperature**	1	0.66014	1.7036	0.008	1	0.58418	1.3933	0.005
**DO**	1	0.49660	1.2815	0.090	1	0.48560	1.1582	0.075
**Bac1**	1	0.35004	0.9033	0.711	na	na	na	na
**Bac2**	1	0.48701	1.2568	0.096	na	na	na	na
**Arc1**	na	na	na	na	1	0.46535	1.1099	0.130
**Arc2**	na	na	na	na	1	0.48275	1.1514	0.069
**Residual**	3	1.16253			3	1.25783		
