# Supplementary figures and images for: From Recharge, to Groundwater, to Discharge Areas in Aquifer Systems in Quebec (Canada): Shaping of Microbial Diversity and Community Structure by Environmental Factors
Source: Genes (Basel). 2022 Dec 20;14(1):1. doi: 10.3390/genes14010001 (PMC9858702; doi:10.3390/genes14010001)

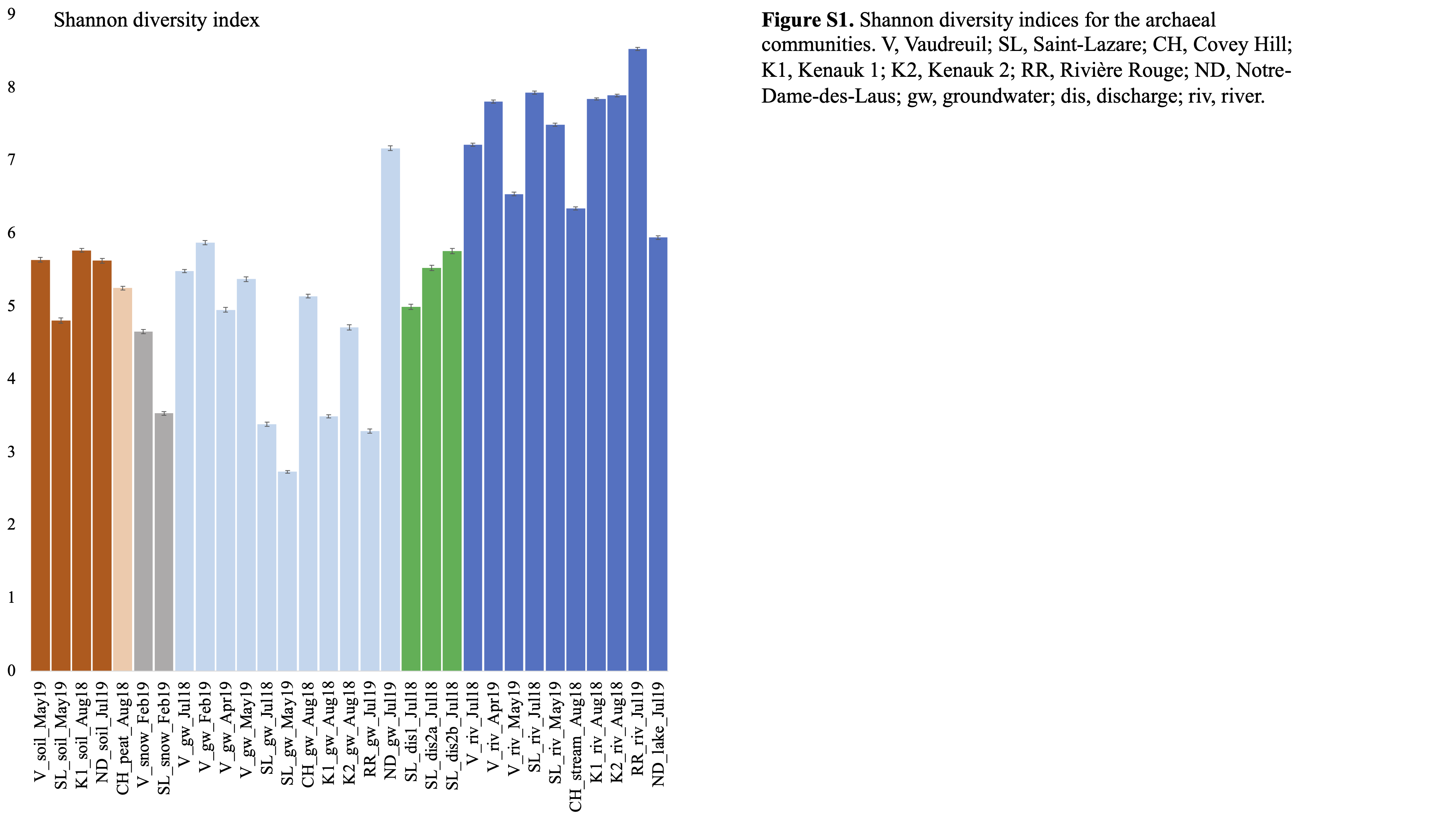

Supplement: Supplementary file 1 [file genes-14-00001-s001.zip › Figure S1.tiff]

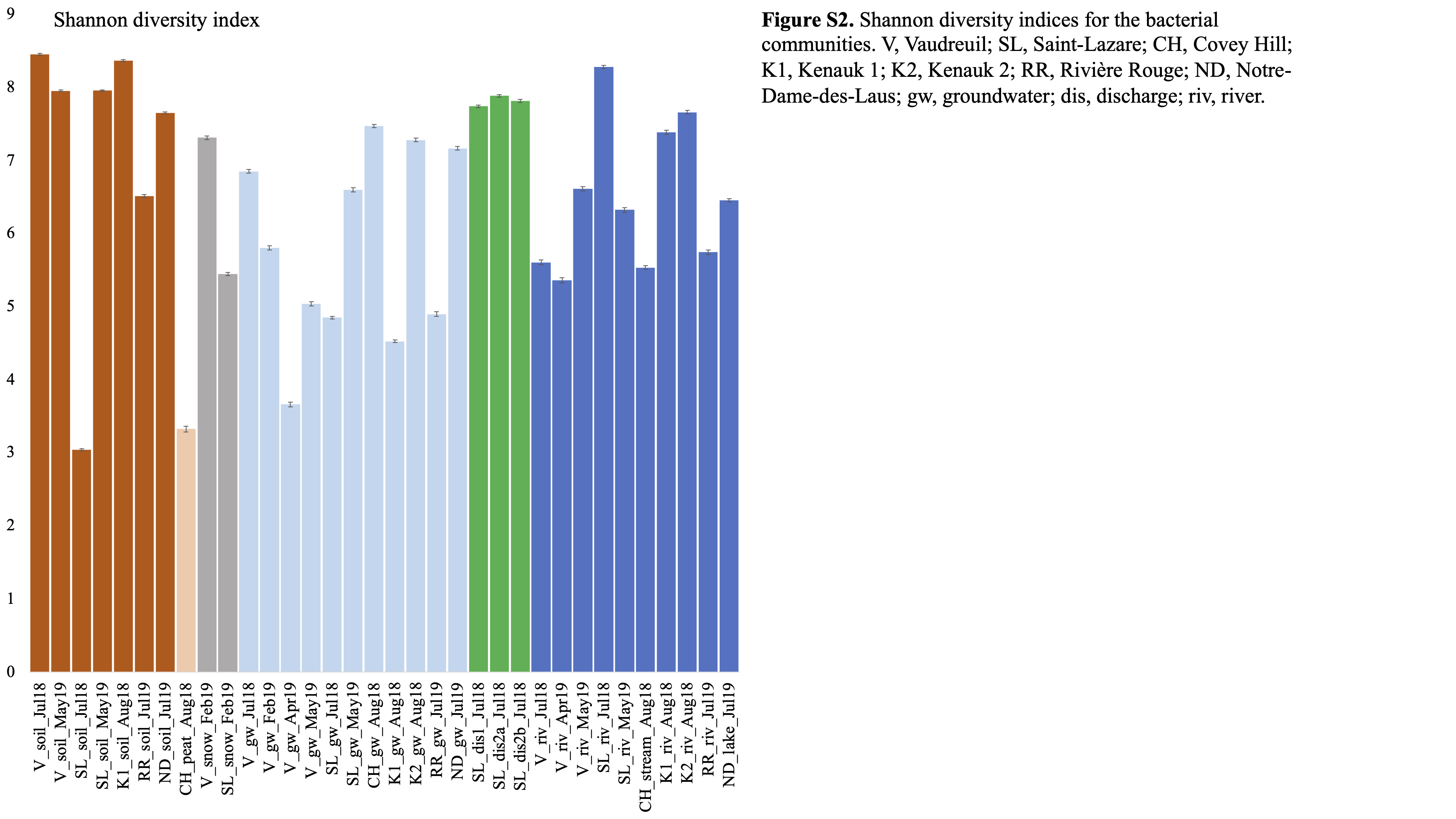

Supplement: Supplementary file 1 [file genes-14-00001-s001.zip › Figure S2.tiff]

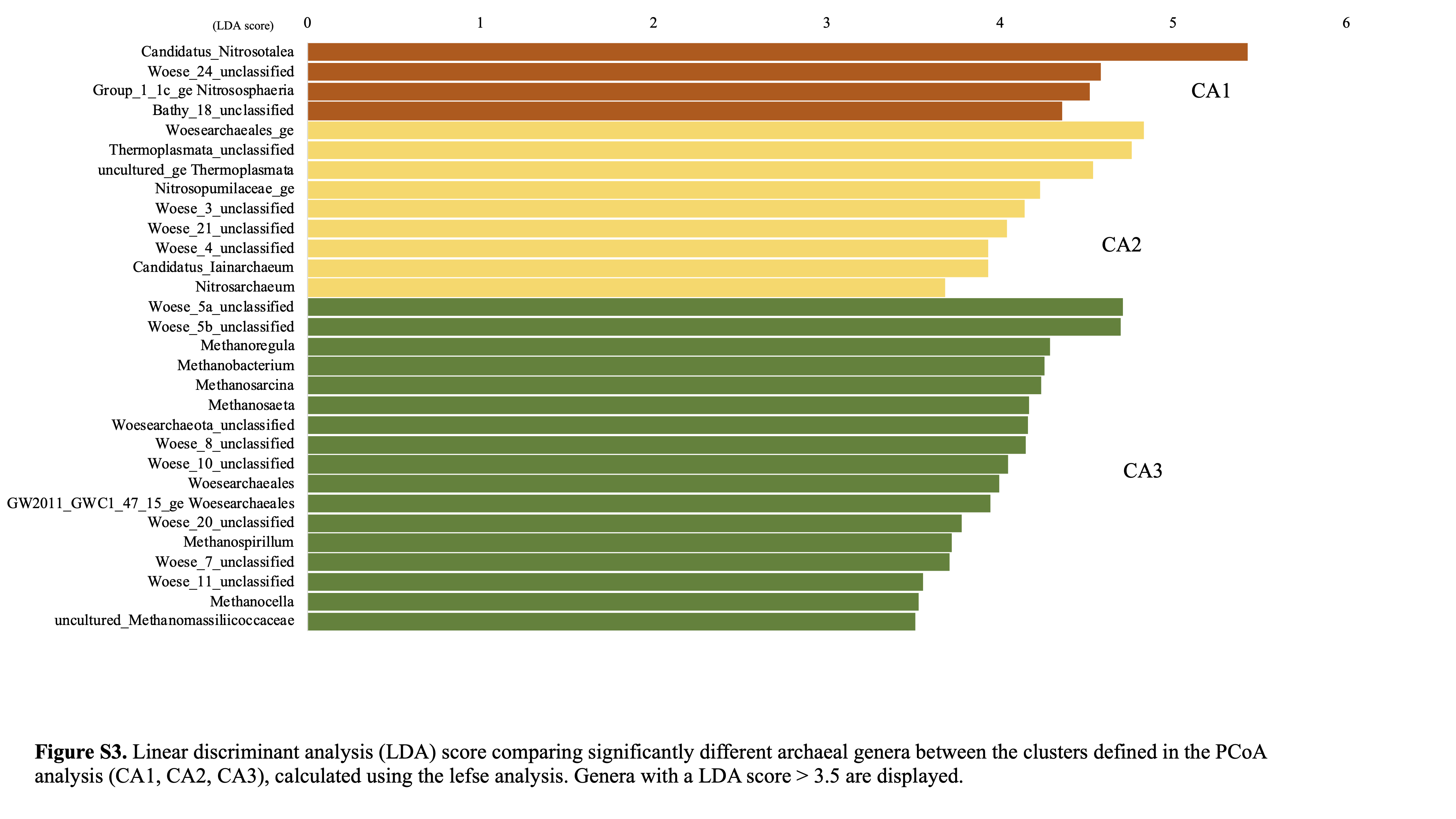

Supplement: Supplementary file 1 [file genes-14-00001-s001.zip › Figure S3.tiff]

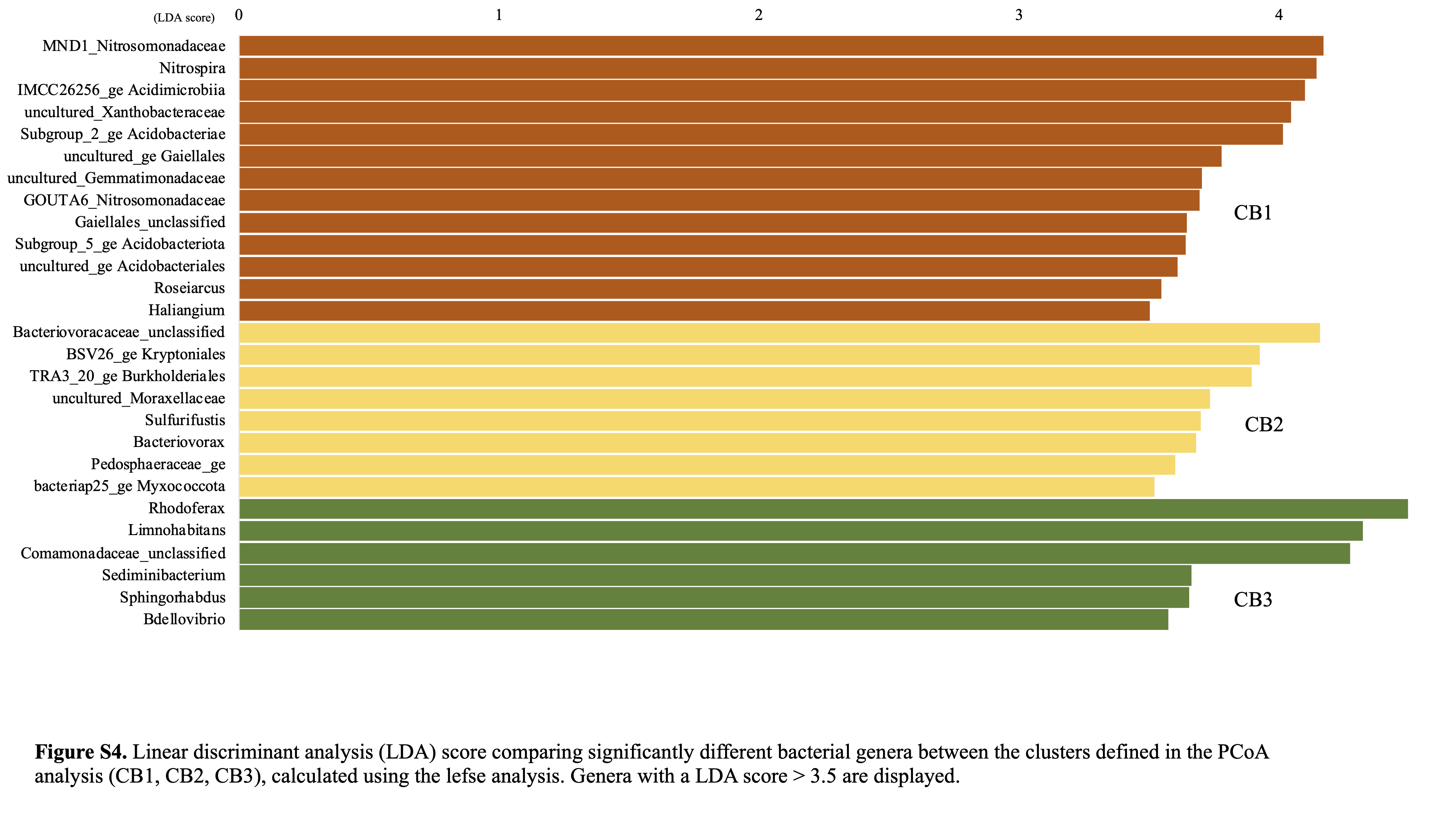

Supplement: Supplementary file 1 [file genes-14-00001-s001.zip › Figure S4.tiff]

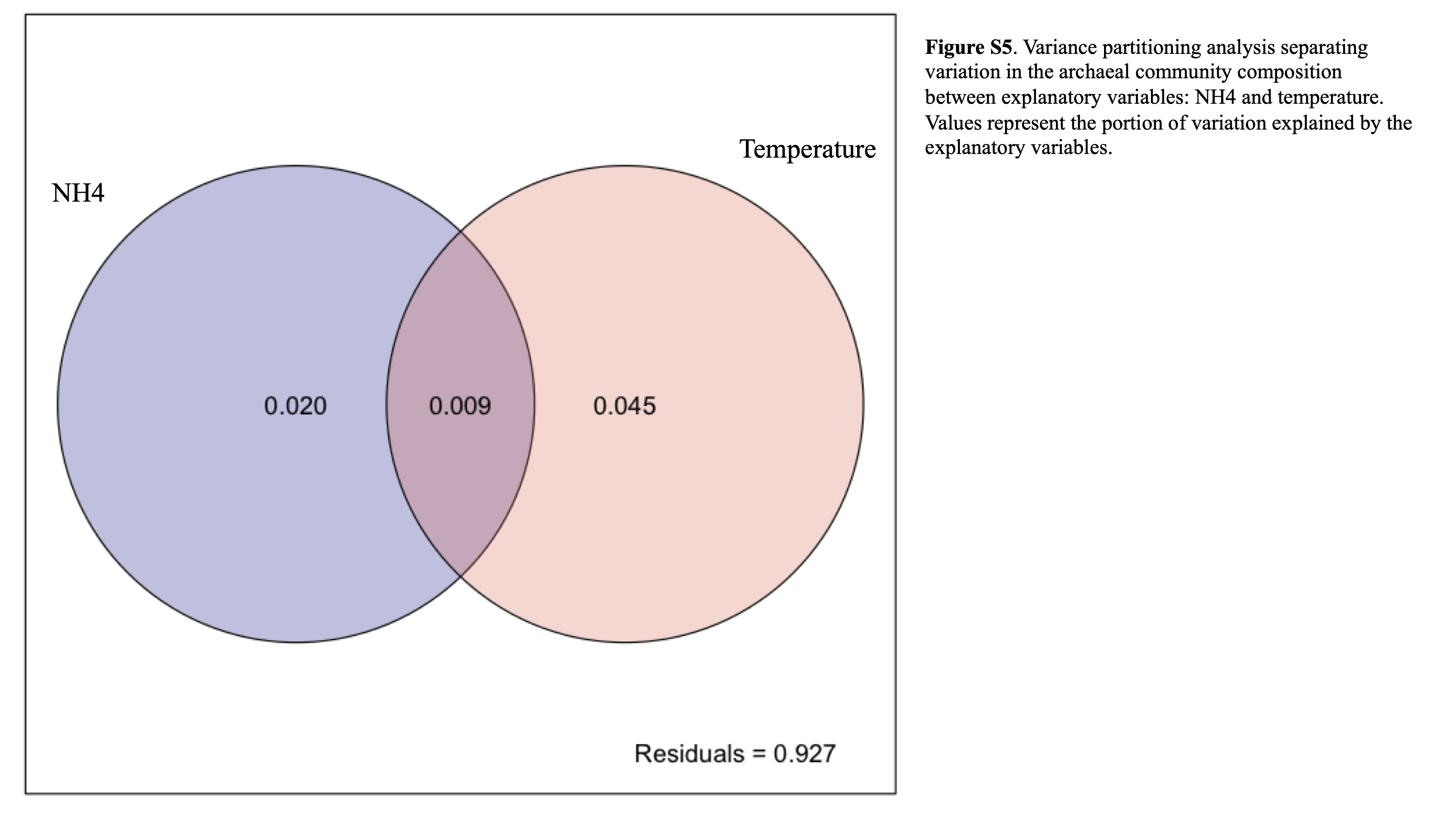

Supplement: Supplementary file 1 [file genes-14-00001-s001.zip › Figure S5.tiff]

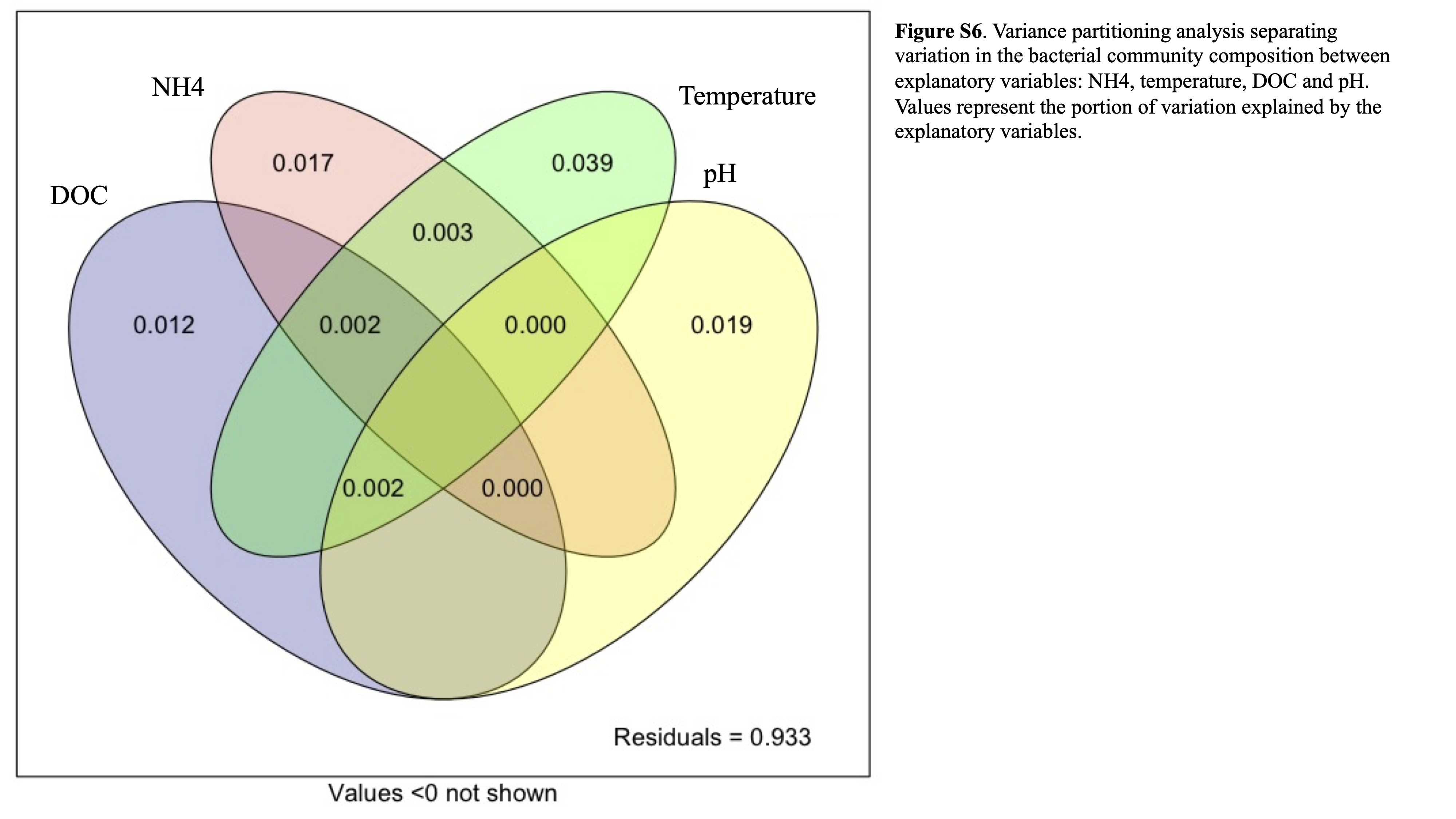

Supplement: Supplementary file 1 [file genes-14-00001-s001.zip › Figure S6.tiff]
